# Cancer metastasis: issues and challenges

**DOI:** 10.1186/s40880-017-0206-7

**Published:** 2017-04-03

**Authors:** Chao-Nan Qian, Yan Mei, Jian Zhang

**Affiliations:** 1 State Key Laboratory of Oncology in South China; Collaborative Innovation Center for Cancer Medicine, Sun Yat-sen University Cancer Center, Guangzhou, 510060 Guangdong P. R. China; 2 Center for Translational Medicine, Guangxi Medical University, and Key Laboratory of Longevity and Aging-related Diseases, Ministry of Education, Nanning, 530021 Guangxi P. R. China; 3 Department of Biology, Southern University of Science and Technology School of Medicine, Shenzhen, 518055 Guangdong P. R. China; 4 Department of Urology, University of Pittsburgh School of Medicine, Pittsburgh, PA 15232 USA

**Keywords:** Metastasis, E-cadherin, EMT, JAM2, PPARGC1A, SIK2, TRAF6

## Abstract

Metastasis is the major cause of treatment failure in cancer patients and of cancer-related deaths. This editorial discusses how cancer metastasis may be better perceived and controlled. Based on big-data analyses, a collection of 150 important pro-metastatic genes was studied. Using The Cancer Genome Atlas datasets to re-analyze the effect of some previously reported metastatic genes—e.g., *JAM2*, *PPARGC1A*, *SIK2*, and *TRAF6*—on overall survival of patients with renal and liver cancers, we found that these genes are actually protective factors for patients with cancer. The role of epithelial–mesenchymal transition (EMT) in single-cell metastasis has been well-documented. However, in metastasis caused by cancer cell clusters, EMT may not be necessary. A novel role of epithelial marker E-cadherin, as a sensitizer for chemoresistant prostate cancer cells by inhibiting Notch signaling, has been found. This editorial also discusses the obstacles for developing anti-metastatic drugs, including the lack of high-throughput technologies for identifying metastasis inhibitors, less application of animal models in the pre-clinical evaluation of the leading compounds, and the need for adjustments in clinical trial design to better reflect the anti-metastatic efficacy of new drugs. We are confident that by developing more effective high-throughput technologies to identify metastasis inhibitors, we can better predict, prevent, and treat cancer metastasis.

Metastasis is the primary cause of cancer-related death [1]. Even in tumors that are sensitive to radiotherapy or chemotherapy, metastasis is often the main reason of treatment failure [2]. Metastatic tumors not only are difficult to treat with conventional surgery or radiotherapy due to their anatomically diffuse localization in different organs, but also, in most cases, are resistant to cytotoxic agents. Although some technological advances in the twentieth century in imaging and cancer cell identification have dramatically improved our understanding of cancer metastasis, the molecular mechanisms underlying cancer metastasis and chemoresistance are mostly unknown. Consequently, of over 200 anti-cancer drugs that have been approved for clinical administration, none specifically and effectively inhibits cancer metastasis.

The mechanisms underlying cancer metastasis are extremely complicated and involve multiple cell types and several key signaling pathways [1]. A review focusing on the interactions between immune cells and tumor cells can be found in this issue of the *Chinese Journal of Cancer* (*CJC*) [3]. More comprehensive discussion of the effect of tumor microenvironment on cancer metastasis can be found in another review article, also in this issue of the *CJC* [4].

Recently, accelerating explorations in the field of cancer metastasis have revealed over 200 genes, in a variety of experimental scenarios, which promote cancer cell motility. A review article in this issue of the *CJC* discusses a collection of 150 important pro-metastatic genes and may be useful for big-data analyses [5]. However, before we can better perceive and more effectively inhibit cancer metastasis, some critical issues must be better understood.

## Pro-metastatic genes do not always associate with poor prognosis

**Fig. 1:**
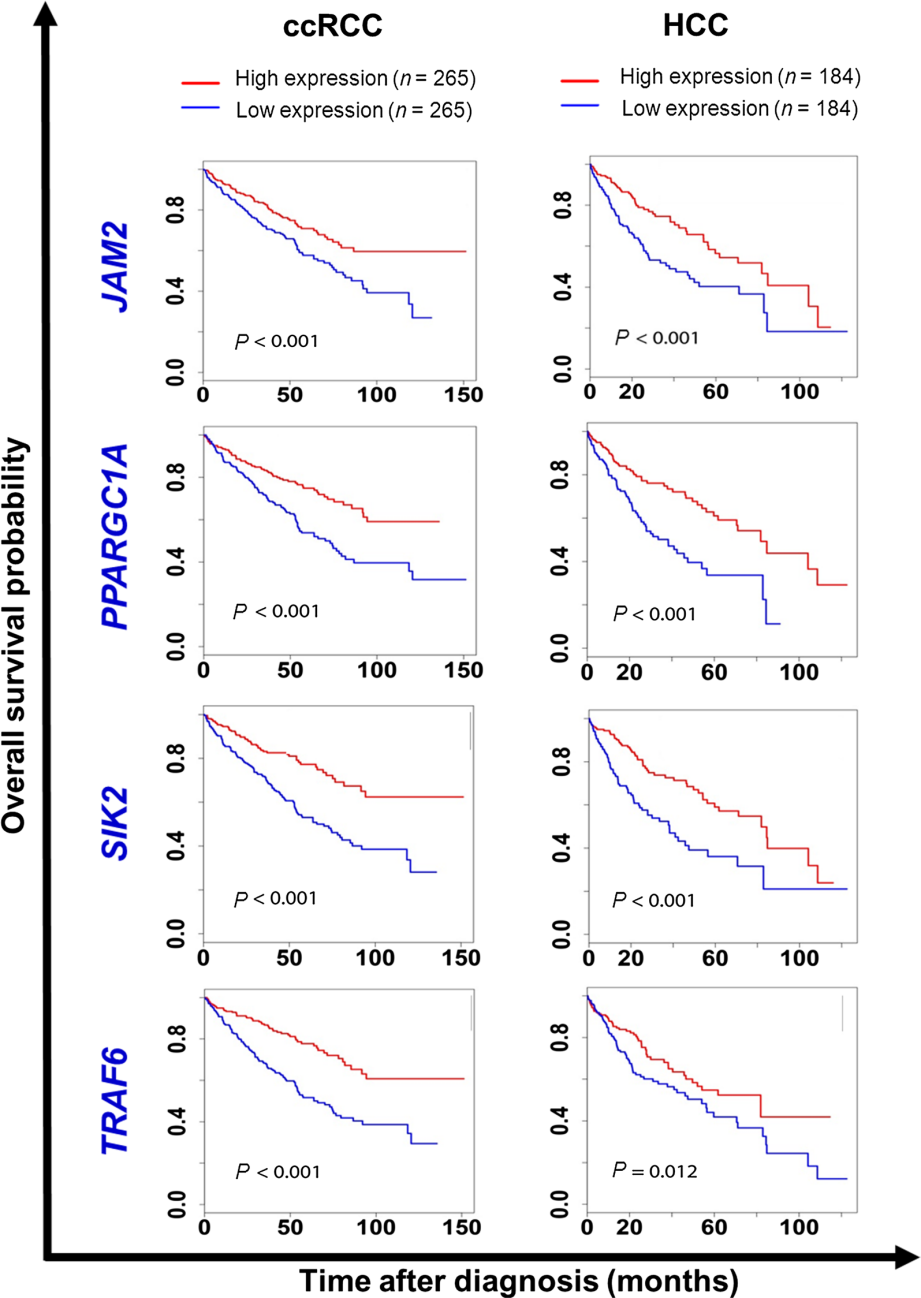
Survival curves of two cohorts of cancer patients separated by the mRNA levels of four genes. The data were retrieved from The Cancer Genome Atlas database. The survival curves were plotted using the Kaplan–Meier method and compared using the log-rank test. The median values were used as cutoff values to separate patients into high- or low-expression groups. *ccRCC* clear cell renal cell carcinoma, *HCC* hepatocellular carcinoma

When we analyzed the pro-metastatic genes using The Cancer Genome Atlas (TCGA) datasets [5], we found that four genes—*JAM2*, *PPARGC1A*, *SIK2*, and *TRAF6*—have contradictory effects on patient overall survival in different types of cancer. In the original reports of these genes’ pro-metastatic functions, *JAM2* was studied in melanoma [6], *PPARGC1A* [7] and *TRAF6* [8] were studied in breast cancer, and *SIK2* was studied in ovarian cancer [9]. When we analyzed the clear cell renal cell carcinoma and hepatocellular carcinoma patient cohorts to evaluate the prognostic value of mRNA levels of the four genes, we found that elevated expression was significantly associated with long overall survival (Fig. 1). Clearly, these four genes encode protective factors for patients with renal cancer and liver cancer. These findings suggest that more complicated mechanisms might underlie cancer metastasis in different tumor types. Furthermore, previous evaluation approaches might not always be appropriate for identifying the true causes of cancer metastasis.

## Issues in developing anti-metastatic drugs

Over 200 anti-cancer drugs have been approved for clinical application. These drugs inhibit tumor cell proliferation, inhibit tumor angiogenesis, or enhance immune function. In the era of targeted therapy, some anti-cancer drugs have multiple effects, such as inhibiting tumor cell proliferation and tumor angiogenesis. Expectations have been high for personalized medicine, which uses genomic information to guide drug application; however, so far for most solid cancer patients, limited survival benefits have been achieved [10, 11]. The primary reason is that metastasis kills most of patients, and very few of these drugs inhibit cancer metastasis.

From a pharmaceutical point of view, many approaches to evaluate the anti-proliferation properties of candidate compounds have been applied with widely accepted standards, from screening the compound using 50% inhibitory concentration (IC_50_) in vitro to measuring tumor growth inhibition rate in vivo. These standardized pharmacologic tests have been routinely required by the U.S. Food and Drug Administration (FDA) for pre-clinical evaluation. However, the dilemma is that the FDA does not require that the drugs under approval show any anti-metastatic effects.

Multiple obstacles hamper the development of anti-metastatic drugs. First, pharmaceutical companies have few effective, high-throughput technologies to screen their compound library for potential anti-metastatic compounds. A recent pioneering study by Polireddy et al. [12] using E-cadherin as a marker to identify inhibitors of cancer cell invasion was a credible attempt to address this issue. Second, most pharmaceutical companies have not developed standard techniques for using appropriate animal models for pre-clinical anti-metastatic assessment, whereas these models are well-established in academic laboratories [13–16]. Third, to better enable the development of anti-metastatic drugs, clinical trial designs might need to be adjusted to set metastasis-free survival as the primary endpoint. Obviously, more efforts are needed to overcome these obstacles, and high-throughput technologies should be developed and optimized to speed the pace of anti-metastatic drug development.

## Single-cell metastasis and epithelial–mesenchymal transition

Epithelial–mesenchymal transition (EMT) has been recognized as an important event to strengthen the metastatic ability of cancer cells. A review article discusses the functions of MTA3 as a master suppressor of EMT and metastasis [17]. E-cadherin has been widely used as an epithelial marker in evaluating EMT. In this issue of the *CJC*, a novel role of E-cadherin as a sensitizer of chemoresistant prostate cancer cells by inhibiting Notch signaling is revealed [18]. However, our understanding of the role of EMT in metastasis is based mainly on the evidence of single-cell motility. When cancer metastasis is caused by cancer cell clusters, EMT of the tumor cells is not necessary [19, 20]. In terms of forming metastatic lesions, cancer cell clusters are more aggressive than single cancer cells [21]. Moreover, cancer cell clusters in circulation can better survive when they are enveloped by endothelial cells [22]. Obviously, more appropriate in vitro and in vivo models for studying cell cluster mobility and metastasis should be developed.

In summary, better prevention and inhibition of cancer metastasis is currently limited by our insufficient understanding of its nature. However, we are confident that through multidisciplinary efforts and the development of more effective, high-throughput technologies to identify metastasis inhibitors, we can better predict, prevent, and treat cancer metastasis.

## References

[1] Steeg PS (2016). Targeting metastasis. Nat Rev Cancer.

[2] Qiu WZ, Huang PY, Shi JL, Xia HQ, Zhao C, Cao KJ (2016). Neoadjuvant chemotherapy plus intensity-modulated radiotherapy versus concurrent chemoradiotherapy plus adjuvant chemotherapy for the treatment of locoregionally advanced nasopharyngeal carcinoma: a retrospective controlled study. Chin J Cancer.

[3] Dai J, Lu Y, Roca H, Keller JM, Zhang J, McCauley LK (2017). Immune mediators in the tumor microenvironment of prostate cancer. Chin J Cancer.

[4] Xie HY, Shao ZM, Li DQ (2017). Tumor microenvironment: driving forces and potential therapeutic targets for breast cancer metastasis. Chin J Cancer.

[5] Mei Y, Yang JP, Qian CN (2017). For robust big data analyses: a collection of 150 important pro-metastatic genes. Chin J Cancer.

[6] Arcangeli ML, Frontera V, Bardin F, Thomassin J, Chetaille B, Adams S (2012). The junctional adhesion molecule-B regulates JAM-C-dependent melanoma cell metastasis. FEBS Lett.

[7] LeBleu VS, O’Connell JT, Gonzalez Herrera KN, Wikman H, Pantel K, Haigis MC (2014). PGC-1α mediates mitochondrial biogenesis and oxidative phosphorylation in cancer cells to promote metastasis. Nat Cell Biol.

[8] Lin Y, Qiu Y, Xu C, Liu Q, Peng B, Kaufmann GF (2014). Functional role of asparaginyl endopeptidase ubiquitination by TRAF6 in tumor invasion and metastasis. J Natl Cancer Inst..

[9] Miranda F, Mannion D, Liu S, Zheng Y, Mangala LS, Redondo C (2016). Salt-inducible kinase 2 couples ovarian cancer cell metabolism with survival at the adipocyte-rich metastatic niche. Cancer Cell.

[10] Perspective Prasad V (2016). The precision-oncology illusion. Nature.

[11] Tannock IF, Hickman JA (2016). Limits to personalized cancer medicine. N Engl J Med.

[12] Polireddy K, Dong R, McDonald PR, Wang T, Luke B, Chen P (2016). Targeting epithelial–mesenchymal transition for identification of inhibitors for pancreatic cancer cell invasion and tumor spheres formation. PLoS ONE.

[13] Li XJ, Ong CK, Cao Y, Xiang YQ, Shao JY, Ooi A (2011). Serglycin is a theranostic target in nasopharyngeal carcinoma that promotes metastasis. Cancer Res.

[14] Li XJ, Peng LX, Shao JY, Lu WH, Zhang JX, Chen S (2012). As an independent unfavorable prognostic factor, IL-8 promotes metastasis of nasopharyngeal carcinoma through induction of epithelial–mesenchymal transition and activation of AKT signaling. Carcinogenesis.

[15] Wang MY, Lin ZR, Cao Y, Zheng LS, Peng LX, Sun R (2016). PDZ binding kinase (PBK) is a theranostic target for nasopharyngeal carcinoma: driving tumor growth via ROS signaling and correlating with patient survival. Oncotarget.

[16] Zheng LS, Yang JP, Cao Y, Peng LX, Sun R, Xie P (2016). SPINK6 promotes metastasis of nasopharyngeal carcinoma via binding and activation of epithelial growth factor receptor. Cancer Res.

[17] Du L, Ning Z, Liu F, Zhang H (2017). Corepressor metastasis-associated protein 3 modulates epithelial-to-mesenchymal transition and metastasis. Chin J Cancer.

[18] Wang W, Wang L, Mizokami A, Shi J, Zou C, Dai J, Keller ET, Lu Y, Zhang J (2017). Down-regulation of E-cadherin enhances prostate cancer chemoresistance via Notch signaling. Chin J Cancer.

[19] Fang JH, Zhou HC, Zhang C, Shang LR, Zhang L, Xu J (2015). A novel vascular pattern promotes metastasis of hepatocellular carcinoma in an epithelial–mesenchymal transition-independent manner. Hepatology.

[20] Lu L, Zeng H, Gu X, Ma W (2015). Circulating tumor cell clusters-associated gene plakoglobin and breast cancer survival. Breast Cancer Res Treat.

[21] Aceto N, Bardia A, Miyamoto DT, Donaldson MC, Wittner BS, Spencer JA (2014). Circulating tumor cell clusters are oligoclonal precursors of breast cancer metastasis. Cell.

[22] Ding T, Xu J, Zhang Y, Guo RP, Wu WC, Zhang SD (2011). Endothelium-coated tumor clusters are associated with poor prognosis and micrometastasis of hepatocellular carcinoma after resection. Cancer.

